# Supplementary material for: Remodeling of the ribosomal quality control and integrated stress response by viral ubiquitin deconjugases
Source: Nat Commun. 2023 Dec 14;14:8315. doi: 10.1038/s41467-023-43946-0 (PMC10721647; doi:10.1038/s41467-023-43946-0)

SUPPLEMENTARY INFORMATION

Supplementary Table 1. Predicted G-quadruplex forming sequences in EBV ORFs[a].

GENE	QGRS	Strand	Expression	Function	Representative sequence[b]	Position (nt)
EBNA1	142	+	latent	Nuclear antigen 1	GGGGCAGGAGCAGGAGGAGG	95965-95984
LMP1	1	-	latent	Latent membrane protein 1	GGGGGGGTCCTCGAGGGGGCCGTCGCGGG	168984-168956
BXLF1	2	-	early	Thymidine kinase	GGCGAGGCATGGAAGGG	131556-131540
BMRF1	1	+	early	DNA polymerase processivity subunit	GGCCGCCGTGGCCAACGCAGGCACGGCCGG	67751-67780
BGLF5	1	-	early	Alkaline exonuclease	GGTGTGAGGACACGGTCAAGG	109563-109543
BdRF1	2	+	late	Small capsid protein, VCA p40	GGGCCCGCGGGCGGGGACCCGGG	136691-136713

a. Putative Quadruplex forming G-Rich Sequences (QGRS) were obtained from both the + and – strands of the EBV B95-8 strain (accession number NC_007605) using the QGRS MAPPER software. A scoring system based on previous knowledge of the known QGRS characteristics, such as size of the loop, symmetry, and number of G tetrads, was applied using the parameters: QGRS max length = 30; minimum G-Group Size = 2; loop size from 0 to 36. A total of 1814 (+ strand) and 1878 (- strand) non-overlapping QGRS were identified. Sequences with a G score lower than 20 were omitted from the analysis.

Supplementary Table 2. PCR and qPCR primers.

Gene	Forward primer(5´-3´)	Reverse primer(5´-3´)
MLN51 qPCR	CAAGGAAGGTCGTGCTGGTT	ACCAGACCGGCCAC CAT
BPLF1 qPCR	CATACACCGTGCGAAAAGAA	GATGGCGGGTAATA CATGCT
EBNA1 qPCR	GGCAGTGGACCTCAAAGAAG	CTATGTCTTGGCCCT GATCC
BMRF1 qPCR	TGACAAGCTTATGGAAACCACT CAGACTCTCC	TCGATGAATTCTTAA ATGAG GGGGTTAAAGGCC
LytLMP1 qPCR	CAGCACAATTCCAAGGAACA	TTTCCCCAGTCACCC TCCT
BXLF1 qPCR	AGGCTGCCGGACATCAAT	TTCTGTGCACGAAG TTTTGC
BPLF1^{C61A} mutagenesis	CGGCATCCAGGCAGTCAGCAAC TGC	GCAAAGCGGCCAAA CTTG
GFP-nonSTOP cloning	CGATCGACTAGTACGCGTGTT ACAAATAAAGCAATAGCATCA	ACGCGTACTAGTCG ATCGCTTGTACAGCT CGTCCATG

Supplementary figures

Supplementary Figure 1. Inhibition of translation elongation by treatment with ANS induces ribosome stalling and collision with consequent RQC activation and ZAKα phosphorylation. HeLa cells were treated with the indicated concentrations of anisomycin (ANS) for 20 min prior to harvesting. Total cell lysates were analyzed by immunoblot using the indicated antibodies. In accordance with published data, maximal RQC activation, assessed by ubiquitination of the 40S ribosome proteins RPS10 and RPS20, and ZAKα phosphorylation, assessed by slower migration, were observed at ANS doses of 0.2-0.5 μg/ml that induce random ribosome stalling and collision. The effects disappeared at higher ANS doses due to a general block of translation.

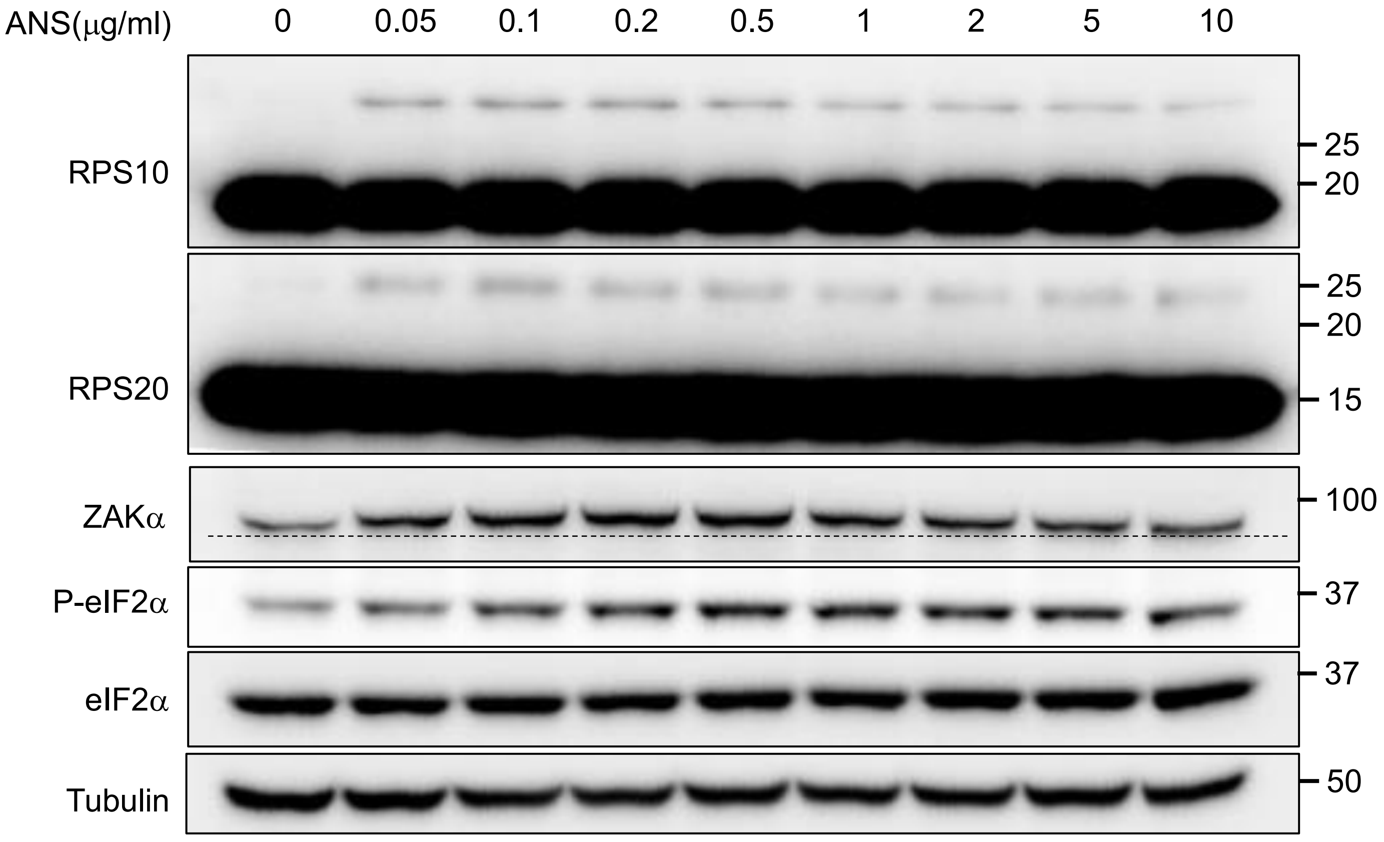

Supplementary Figure 2. Characterization of the HEK293-ZNF598-KO cell line. (**a**) A ZNF598 negative subline of HEK293T was produced by CRISPR/Cas9 knockout. The parental and KO cell lines were treated with 0.5 μg/ml ANS for the indicated time, and western blots were probed with the indicated antibodies. Lack of ZNF598 was associated with failure to induce the ubiquitination of 40S ribosome proteins, while persistent ribosome stalling promoted the phosphorylation of ZAKα, phosphorylation of eIF2α, and the accumulation of ATF4. Blots from one representative experiment out of two are shown in the figure. **(b)** The ZAKα migration shift observed in ANS-treated cells is phosphorylation-dependent. Aliquots of HEK293T cells treated for 30 min with 0.5 μg/ml ANS were lysed in NP40 lysis buffer with or without the addition of a phosphatase inhibitors cocktail. Untreated HEK293T cells were included as a control. A western blot of one representative out of four independent experiments is shown in the figure.

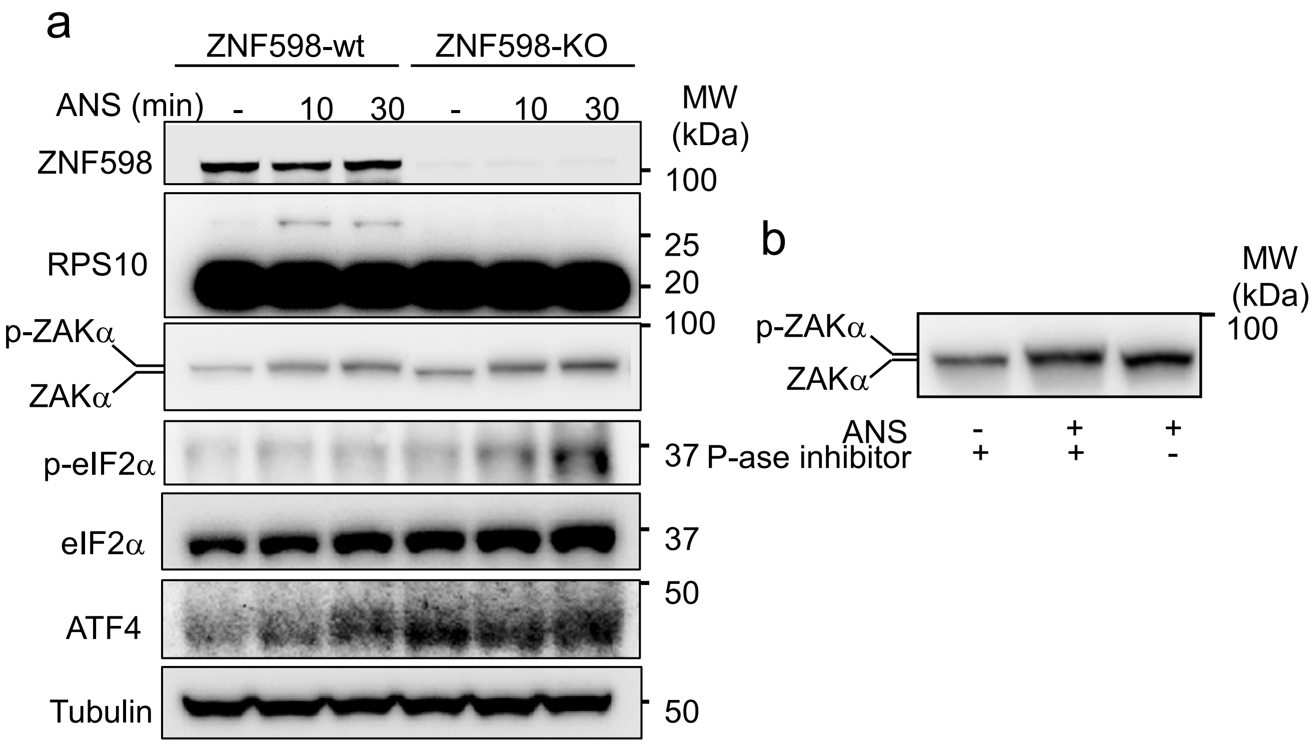

Supplementary Figure 3. The GFPnonSTOP reporter is stabilized in ZNF598-KO cells. Control and HEK293-ZNF598-KO cells were transfected with the GFPnonSTOP reporter, and the reporter's expression levels were monitored after 24 h by probing western blots of total cell lysates with the GFP antibody. The reporter was stabilized in ZNF598-KO cells at levels slightly lower compared to those achieved in control cells by treatment with MG132 or transfection of catalytically active BPLF1, which is likely due to synergistic effects of substrate deubiquitination and inhibition of the proteasome.

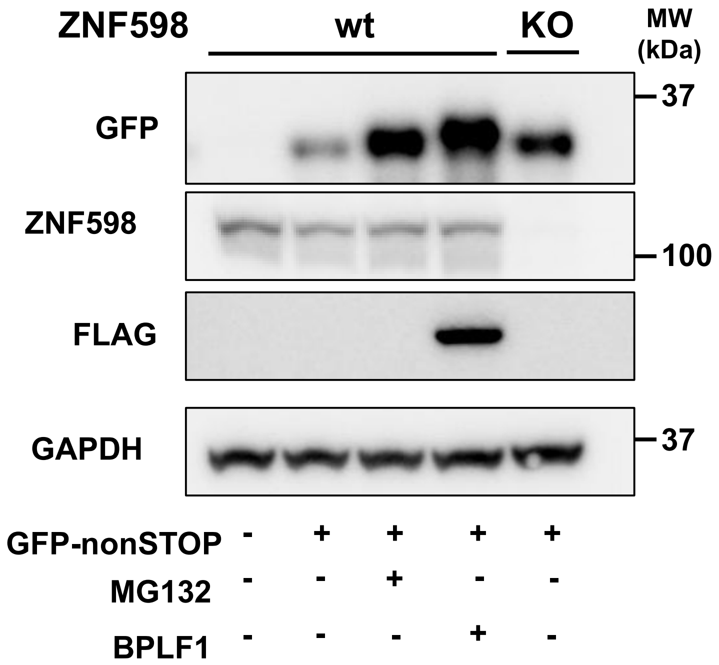

Supplementary Figure 4. BPLF1 promotes the readthrough of stall-inducing mRNA at a level comparable to the knockout of ZNF598. Readthrough of the K20 reporter was assessed in control HEK293T cells with and without co-transfection of catalytically active BPLF1 and in ZNF598-KO cells. HEK293T cells transfected with the K0 reporter were included for gating the readthrough (R) region, where the cells exhibit a linear correlation between GFP and RFP fluorescence. Cells falling in the stalling (S) region exhibited decreased RFP:GFP fluorescence ratios. The catalytically active vDUB rescued RFP fluorescence in the K20 expressing cells at levels slightly lower compared to full ZNF598 knockout, which is in line with the expected efficiency of transfection. FACS plots from one representative experiment out of two are shown.

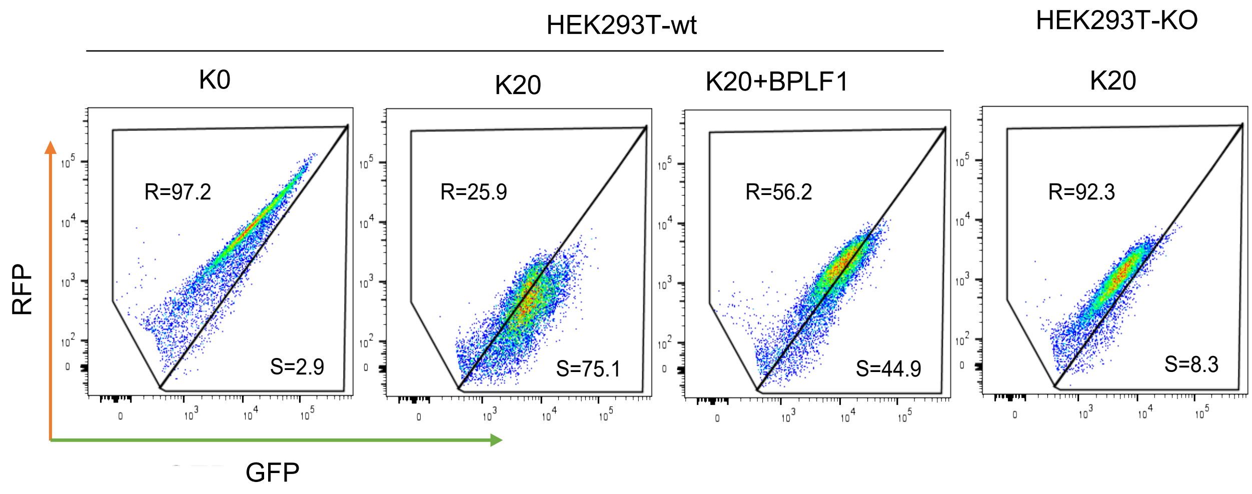

Supplementary Figure 5. Reconstitution of cap-dependent translation by treatment with ISRIB does not affect the inhibition of RQC and stabilization of a model RQC substrate by BPLF1. (**a**) HeLa cells transfected overnight with plasmids expressing FLAG-tagged BPLF1, the BPLF1^{C61A} mutant, or the empty FLAG vector were kept untreated or treated for 2 h with 1 µM ISRIB followed by treatment for 20 min with 0.5 µg/ml ANS. Western blots were probed with the indicated antibodies. Treatment with ISRIB did not affect the capacity of BPLF1 to inhibit the RQC assessed by decreased ubiquitination of RPS10 and RPS20. **(b)** Western blots of HeLa cells transfected with a control GFP plasmid or the GFPnonSTOP reporter alone or with FLAG-BPLF1. Treatment with ISRIB did not promote the accumulation of the reporter and did not alter the stabilization induced by BPLF1.

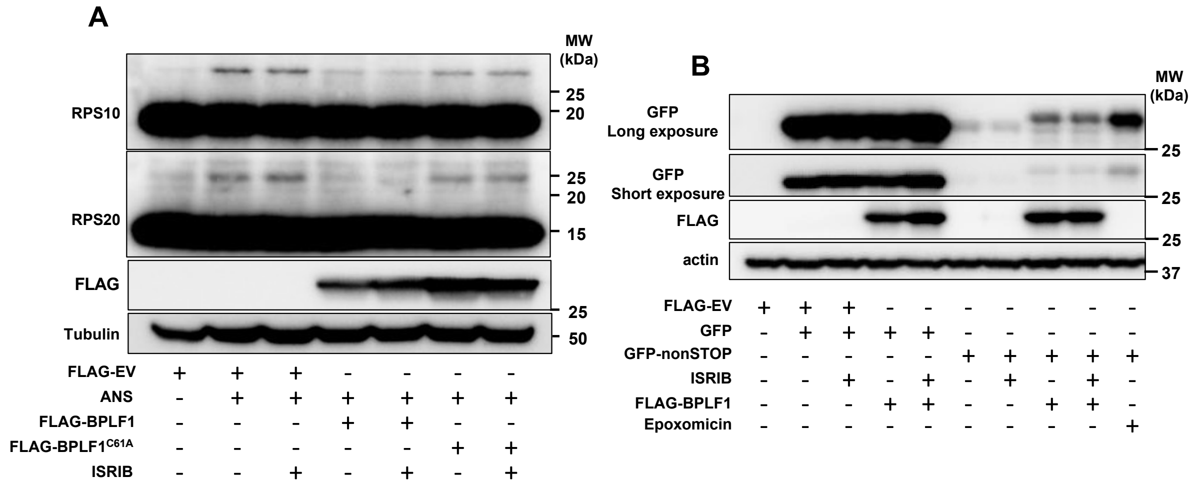

Supplementary Figure 6. Expression of the BZLF1 transactivator in Dox-treated LCLs carrying catalytic active and mutant BPLF1. (**a**) The productive virus cycle was induced in LCL cells carrying wt and mutant BPLF1 and a Dox-regulated BZLF1 transactivator by treatment with 1.5 μg/ml Dox for 72 h. Representative western blot illustrating the comparable expression of the BZLF1 in induced LCL cells. (**b**) Mean ± SD relative BZLF1 intensity in three independent experiments.

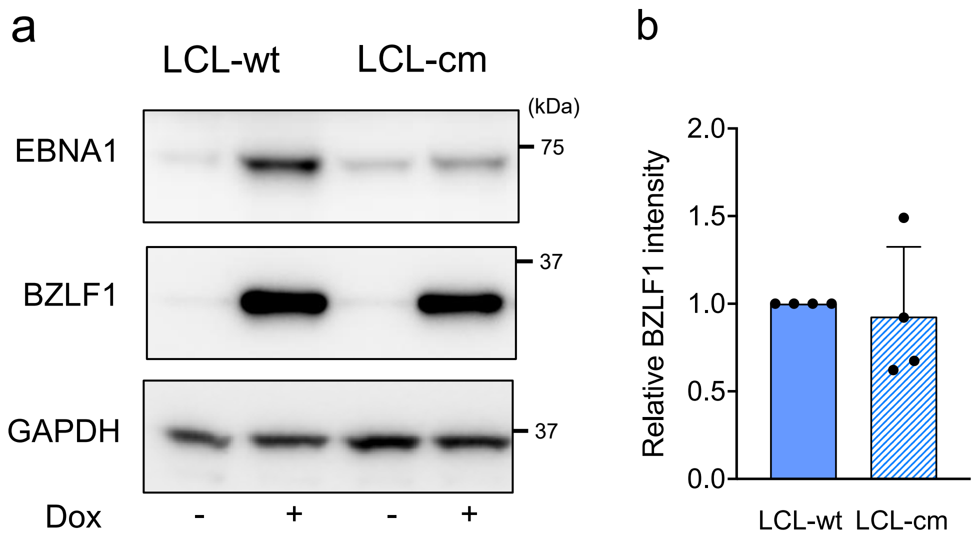

Supplementary Figure 7. Treatment with proteasome inhibitors does not rescue the upregulation of EBNA1 in induced LCLcm cells. The productive virus cycle was induced by treatment with Dox for 72 h in LCLs immortalized with recombinant EBV expressing the wild-type (LCLwt) or catalytic mutant (LCLcm) BPLF1 and transduced with a Dox-regulated BZLF1 transactivator. Aliquots of controls and induced cells were treated with 10 µM MG132 during the last 6 h before harvesting and western blot analysis. Cell lysates were probed with the indicated specific antibodies. Blots from one representative experiment out of two are shown in the figure.

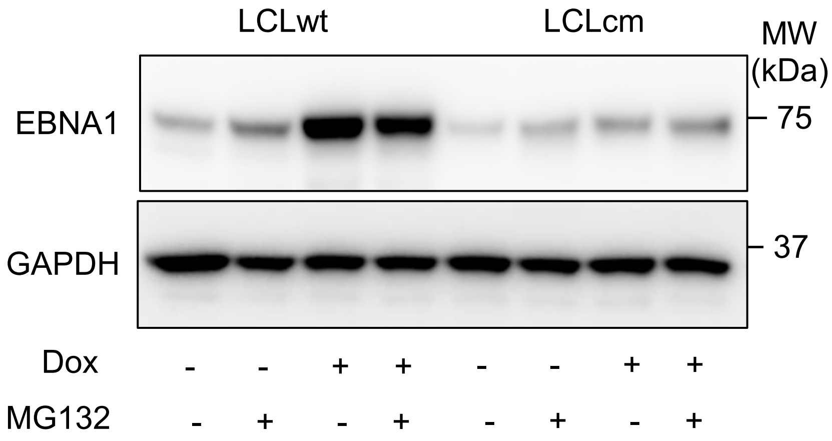

Supplementary Figure 8. Catalytically active BPLF1 promotes the translation of all classes of viral mRNAs during productive EBV infection. (**a**). Catalytically active BPLF1 promotes the expression of a selection of early and late viral proteins. Western blots of cell lysates from the induction experiments shown in Figure 6 were probed with the indicated antibodies. (**b**) Densitometric quantification of protein and mRNA expression in two to four independent experiments. Due to undetectable expression in uninduced cells, protein levels are expressed as the ratio of band intensity in induced cells expressing wild-type and mutant BPLF1. (**c** and **d**) The viral proteins are not proteasomal substrates. Aliquots of controls and induced cells were treated with 10 µM MG132 during the last 6 h before harvesting and western blot analysis.

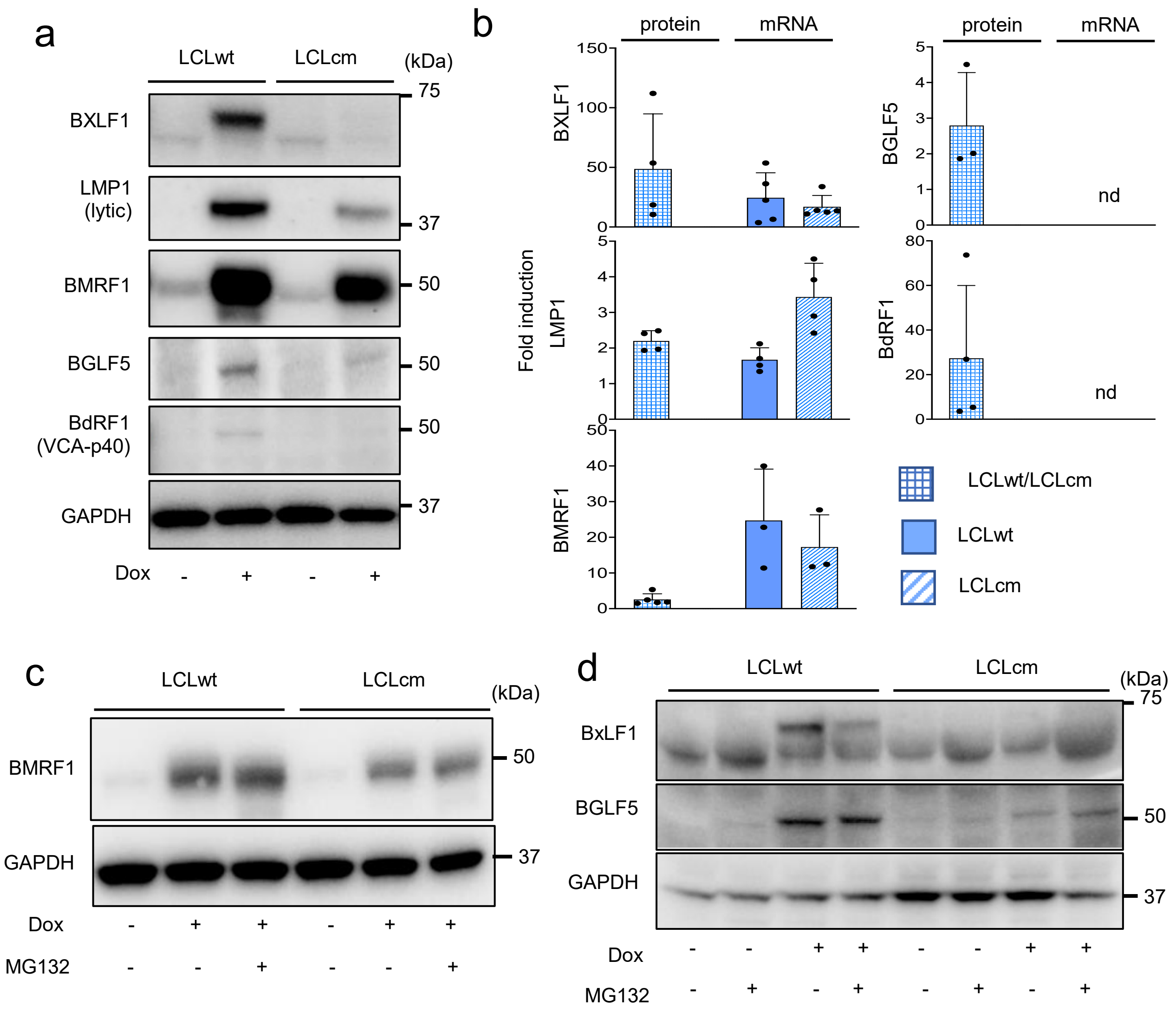

Supplementary Figure 9. The N-terminal domains of the BPLF1 homolog encoded by HSV1, HCMV, and KSHV are active vDUBs. Lysates of HeLa cells transfected for 24 h with the indicated plasmids were incubated with 1 μM of the functional probe Ub-VS that forms covalent bonds with the catalytic Cys residue of active DUBs. After incubation for 1 h at 37°C, the lysates were fractionated by SDS-PAGE, and the viral proteins were detected in western blots probed with the anti-FLAG antibody. DUB activity was confirmed by a shift in molecular weight corresponding to the size of the covalently bound probe (indicated by arrows). The shift was not observed in the lysates of cells expressing catalytic mutant BPLF1^{C61A}.

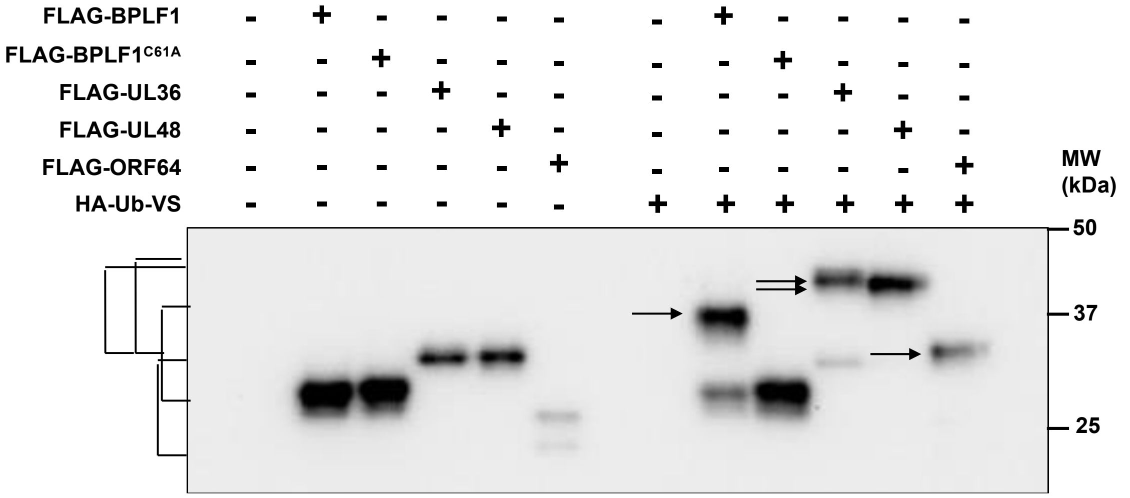

Supplement: Supplementary file 1 — Supplementary Information [file 41467_2023_43946_MOESM1_ESM.pdf]
