# Supplementary material for: Remodeling of the ribosomal quality control and integrated stress response by viral ubiquitin deconjugases
Source: Nat Commun. 2023 Dec 14;14:8315. doi: 10.1038/s41467-023-43946-0 (PMC10721647; doi:10.1038/s41467-023-43946-0)
Supplement: Supplementary file 3 — Description of Additional Supplementary Files [file 41467_2023_43946_MOESM3_ESM.pdf]

Description of Additional Supplementary Files

File name: Supplementary Data 1
Description: Mass Spectrometry data including, number of replicas, conditions of the assay, software used for protein identification, legends
Type of file: Excel

File name: Supplementary Data 2
Description: Functional annotation of the BPLF1 interacting proteins involved in translation and RQC. Curated annotation from the STRING database v 9.0
Type of file: Excel

File Name Supplementary Data 3
Description: List of reagents used in the study, including source, identifier, catalog number, and working conditions
Type of file: Excel
