# Supplementary material for: Remodeling of the ribosomal quality control and integrated stress response by viral ubiquitin deconjugases
Source: Nat Commun. 2023 Dec 14;14:8315. doi: 10.1038/s41467-023-43946-0 (PMC10721647; doi:10.1038/s41467-023-43946-0)
Supplement: Supplementary file 7 — Reporting Summary [file 41467_2023_43946_MOESM7_ESM.pdf]

nature portfolio

Corresponding author(s): Maria Grazia Masucci

Last updated by author(s): Nov 7, 2023

Reporting Summary

Nature Portfolio wishes to improve the reproducibility of the work that we publish. This form provides structure for consistency and transparency in reporting. For further information on Nature Portfolio policies, see our Editorial Policies and the Editorial Policy Checklist.

Statistics

For all statistical analyses, confirm that the following items are present in the figure legend, table legend, main text, or Methods section.

n/a	Confirmed	
☐	☒	The exact sample size (*n*) for each experimental group/condition, given as a discrete number and unit of measurement
☐	☒	A statement on whether measurements were taken from distinct samples or whether the same sample was measured repeatedly
☐	☒	The statistical test(s) used AND whether they are one- or two-sided<br>*Only common tests should be described solely by name; describe more complex techniques in the Methods section.*
☒	☐	A description of all covariates tested
☒	☐	A description of any assumptions or corrections, such as tests of normality and adjustment for multiple comparisons
☐	☒	A full description of the statistical parameters including central tendency (e.g. means) or other basic estimates (e.g. regression coefficient) AND variation (e.g. standard deviation) or associated estimates of uncertainty (e.g. confidence intervals)
☐	☒	For null hypothesis testing, the test statistic (e.g. *F*, *t*, *r*) with confidence intervals, effect sizes, degrees of freedom and *P* value noted<br>*Give P values as exact values whenever suitable.*
☒	☐	For Bayesian analysis, information on the choice of priors and Markov chain Monte Carlo settings
☒	☐	For hierarchical and complex designs, identification of the appropriate level for tests and full reporting of outcomes
☒	☐	Estimates of effect sizes (e.g. Cohen's *d*, Pearson's *r*), indicating how they were calculated

Our web collection on statistics for biologists contains articles on many of the points above.

Software and code

Policy information about availability of computer code

Data collection	Software form mass spectrometry data collection are specified in Supplementary Data 1. Biorad Image Lab v. 6.0.1 was used for immunoblot data collection. Flow cytometry data were collected with a BD LSR II SORP apparatus. Confocal images were collected with a confocal fluorescence laser scanning Zeiss LSM900 microscope
Data analysis	Mass spectrometry: String: v9.0; DAVID; ToppCluster; Gene Ontology; Cytoscape: v.3.9.1; Western blot: Image Lab: v.6.0.1 build 34; Confocal images: Fiji imageJ: v.2.1.0/1.53c; Statistics: GraphPad Prism 10: Version 10.1.0 (264); Flow cytoimetry FlowJo: v10.9

For manuscripts utilizing custom algorithms or software that are central to the research but not yet described in published literature, software must be made available to editors and reviewers. We strongly encourage code deposition in a community repository (e.g. GitHub). See the Nature Portfolio guidelines for submitting code & software for further information.

Data

Policy information about availability of data

All manuscripts must include a data availability statement. This statement should provide the following information, where applicable:

- Accession codes, unique identifiers, or web links for publicly available datasets
- A description of any restrictions on data availability
- For clinical datasets or third party data, please ensure that the statement adheres to our policy

Data provided in Supplementary Data 1

Research involving human participants, their data, or biological material

Policy information about studies with human participants or human data. See also policy information about sex, gender (identity/presentation), and sexual orientation and race, ethnicity and racism.

Reporting on sex and gender	EBV transformed LCLs were established from PBMCs purchased from the Blood Bank of Karolinska Hospital. Information on sex and gender is not available and is not relevant to the study
Reporting on race, ethnicity, or other socially relevant groupings	Information on sex, race, and ethnicity is not available for anonimous donor and is not relevant to the study
Population characteristics	N/A
Recruitment	N/A
Ethics oversight	N/A

Note that full information on the approval of the study protocol must also be provided in the manuscript.

Field-specific reporting

Please select the one below that is the best fit for your research. If you are not sure, read the appropriate sections before making your selection.

☒ Life sciences ☐ Behavioural & social sciences ☐ Ecological, evolutionary & environmental sciences

For a reference copy of the document with all sections, see nature.com/documents/nr-reporting-summary-flat.pdf

Life sciences study design

All studies must disclose on these points even when the disclosure is negative.

Sample size	Data were obtained from repeated independent experiments. Each experiments was repeated between two to five time in order to assess reproducibility and obtain statistical data. The number of independent experiments for each condition is specified in Material and methods and figure legends. No sample size calculation was performed.
Data exclusions	No data were excluded from the analysis
Replication	Each experiments was repeated between two to five time to assure reproducibility.
Randomization	No randomization method was used. However, proper controls were included in each experiment
Blinding	Fluorescence assays were independently assessed by the main investigator and by a collaborator who was blinded to group allocation

Reporting for specific materials, systems and methods

We require information from authors about some types of materials, experimental systems and methods used in many studies. Here, indicate whether each material, system or method listed is relevant to your study. If you are not sure if a list item applies to your research, read the appropriate section before selecting a response.

Materials & experimental systems

n/a	Involved in the study	
☐	☒	Antibodies
☐	☒	Eukaryotic cell lines
☒	☐	Palaeontology and archaeology
☒	☐	Animals and other organisms
☒	☐	Clinical data
☒	☐	Dual use research of concern
☒	☐	Plants

Methods

n/a	Involved in the study	
☒	☐	ChIP-seq
☐	☒	Flow cytometry
☒	☐	MRI-based neuroimaging

Antibodies

Antibodies used	Mouse monoclonal anti-β-actin clone AC-15 Sigma-Aldrich Cat# A5441, RRID: AB_476744 1:5000<br>Mouse monoclonal anti-GAPDH Millipore Cat#CB1001 1:5000

Mouse monoclonal anti-tubulin Millipore Cat#CP06 1:2000
Mouse monoclonal anti-FLAG Sigma-Aldrich Cat# F3165, RRID: AB_259529 1:10000
Rabbit polyclonal anti-FLAG Sigma-Aldrich Cat#F7425, RRID: AB_439687 1.10000
Rabbit IgG isotype control Abcam Cat# ab172730, RRID:AB_2687931
Donkey anti-Mouse IgG (H+L), Alexa Fluor 555 Thermo Fisher Scientific Cat# A-31570, RRID: AB_2536180 1.1000
Donkey Anti-Rabbit IgG (H+L) Antibody, Alexa Fluor 488 Thermo Fisher Scientific Cat# A-21206, RRID: AB_2535792 1:1000
Goat Anti-Rabbit IgG (H+L) Antibody, Alexa Fluor 647 Thermo Fisher Scientific Cat#A21245, RRID: AB_141775 1:1000
Rabbit polyclonal anti-ZNF598 Invitrogen Cat# PA559777, RRID: AB_2650226 1:1000
Rabbit polyclonal anti-ZNF598 Abcam AB241092 1:5000
Rabbit polyclonal anti-LTN1 GeneTex Cat# GTX18154 1:3000
Rabbit polyclonal anti-SEC63 Bethyl Laboratories Cat# A305-084A, RRID: AB_2631479 1:1000
Rabbit polyclonal anti-RPN1 Bethyl Laboratories Cat# A305-026A, RRID: AB_2621220 1:1000
Mouse monoclonal anti-RPN1 Santa Cruz Biotechnology Cat# sc-48367, RRID: AB_628221 1:1000
Rabbit polyclonal anti-SRPRB Bethyl Laboratories Cat# A305-440A, RRID: AB_2631831 1:1000
Rabbit polyclonal anti-SRP68 Bethyl Laboratories Cat# A303-955A, RRID: AB_2620304 1:1000
Mouse monoclonal anti-VCP Abcam Cat# ab11433, RRID:AB_298039 1:5000
Rabbit monoclonal anti-RPS10 Abcam Cat# ab151550, RRID:AB_2714147 1.3000
Rabbit monoclonal anti-RPS20 Abcam Cat# ab133776, RRID:AB_2714148 1.2000
Rabbit polyclonal anti-SEC61B Thermo Fisher Scientific Cat# PA3-015 1:10000
Rabbit polyclonal anti-eIF4G2 (DAP5) Bethyl Laboratories Cat# A302-249A, RRID: AB_1730977 1:1000
Mouse monoclonal anti-eIF4G1 (2A9) Abnova Cat# H00001981-M10, RRID: AB_606171 1:1000
Rabbit anti-Phospho-eIF2a-Ser51 (D9G8) XP Cell Signaling Technology Cat# 3398, RRID: AB_2096481 1:1000
Mouse monoclonal anti-eIF2a (L57A5) Cell Signaling Technology Cat#2103, RRID: AB_836874 1:1000
Rabbit monoclonal anti-ATF4 (D4B8) Cell Signaling Technology Cat# 11815, RRID: AB_2616025 1:1000
Mouse monoclonal anti-CHOP (L63F7) Cell Signaling Technology Cat# 2895, RRID: AB_2089254 1:1000
Mouse monoclonal anti-Puromycin (12D10) Millipore Cat# MABE343, RRID: AB_2566826 1:10000
Rabbit polyclonal anti-ZAK Proteintech Cat# 28761-1-AP, RRID:AB_2918199 1:2000
Recombinant rabbit anti-Phospho-JNK (Tyr185) Proteintech Cat# 80024-1-RR 1:1000
Rabbit polyclonal anti-Phospho-p38 MAPK(Thr180/Tyr182) Cell Signaling Technology Cat#9211 1:1000
Rat monoclonal anti-EBV-EBNA1 MAB core facility, Helmholtz Center, Munich, Germany N/A 1:100
Mouse monoclonal anti-EBV-BZLF1 Santa Cruz Biotechnology Cat# sc-53904 1:1000
Rabbit polyclonal anti-EBV-BdRF1 Dr. Jaap M. Middeldorp, VU University Medical Center,Amsterdam, NL N/A 1:1000
Rabbit polyclonal anti-EBV-BGLF5 Dr. Jaap M. Middeldorp, VU University Medical Center,Amsterdam, NL N/A 1:1000
Mouse monoclonal anti LMP1 (CS1-4) Dako, Glostrup, Denmark Cat# M0897 1:1000
Rabbit polyclonal anti-EBV BXLF1 ASLA Biotech, Riga Latvia N/A 1:200
Mouse monoclonal anti-EBV-BMRF1 Dr. Jaap M. Middeldorp, VU University Medical Center,Amsterdam, NL N/A 1:10000

Validation

All antibodies were validated by the manufactures and were previously used in peer reviewed work. Methods of validation are provided in the dedicated website pages. Working conditions for each antibody were assessed in preliminary experiments to confirm detection of the expected size product in western blots and immunofluorescence including appropriated positive and negative controls

Eukaryotic cell lines

Policy information about cell lines and Sex and Gender in Research

Cell line source(s)

A list of the cell lines used with source and references is provided in Supporting information Table 3. Established cell lines were commercially purchased (HeLa and HEK293T, ATCC). Stable sublines (HEK293T- ZNF598-KO, HEK-EBV BPLF1 wt/mut) were produced in house from early passages. EBV immortalized LCLs (LCLwt/cm Tet-on BZLF1) were produced in house from PBMC of anonimous blood bank donors.

Authentication

No further authentication was performed for commercially purchased cell lines

Mycoplasma contamination

All cell lines are mycoplasma free as assessed by regular PCR testing

Commonly misidentified lines
(See ICLAC register)

Early passages of validated commercially available HeLa and HEK293T cell were used

Plants

Seed stocks

N/A

Novel plant genotypes

N/A

Authentication

N/A

Flow Cytometry

Plots

Confirm that:

☒ The axis labels state the marker and fluorochrome used (e.g. CD4-FITC).

☒ The axis scales are clearly visible. Include numbers along axes only for bottom left plot of group (a 'group' is an analysis of identical markers).

☒ All plots are contour plots with outliers or pseudocolor plots.

☒ A numerical value for number of cells or percentage (with statistics) is provided.

Methodology

Sample preparation	Detailed protocols for cell labeling and sample preparation are provided in the methods section
Instrument	A BD LSR 11 SORP apparatus was used
Software	The FlowJo software was used for analysis
Cell population abundance	10000 cells were analyzed for each sample after gating of living cells based on FCS/SSC
Gating strategy	Cell doublets were removed and cells selection was based on forward and side scatter. Single cells were gated based on the GFP versus RFP fluorescence. Further details details of the gating strategy used to identify stalling and read-through cell populations are provided in the text and figures

☐ Tick this box to confirm that a figure exemplifying the gating strategy is provided in the Supplementary Information.
